# Investigation of Persistent Photoconductivity of Gallium Nitride Semiconductor and Differentiation of Primary Neural Stem Cells

**DOI:** 10.3390/molecules29184439

**Published:** 2024-09-19

**Authors:** Yu Meng, Xiaowei Du, Shang Zhou, Jiangting Li, Rongrong Feng, Huaiwei Zhang, Qianhui Xu, Weidong Zhao, Zheng Liu, Haijian Zhong

**Affiliations:** 1 Jiangxi Provincal Key Laboratory of Tissue Engineering, Gannan Medical University, Ganzhou 341000, China; mengyu1@gmu.cn (Y.M.); d1883456@163.com (X.D.); zhoushang@gmu.edu.cn (S.Z.);; 2 School of Medical Information Engineering, Gannan Medical University, Ganzhou 341000, China; 3 Key Laboratory of Prevention and Treatment of Cardiovascular and Cerebrovascular Diseases, Ministry of Education, Gannan Medical University, Ganzhou 341000, China; 4 School of Materials Science & Engineering, Nanyang Technological University, Singapore 639798, Singapore

**Keywords:** GaN, neural stem cells, semiconductor biointerface, Kelvin probe force microscopy

## Abstract

A gallium nitride (GaN) semiconductor is one of the most promising materials integrated into biomedical devices to play the roles of connecting, monitoring, and manipulating the activity of biological components, due to its excellent photoelectric properties, chemical stability, and biocompatibility. In this work, it was found that the photogenerated free charge carriers of the GaN substrate, as an exogenous stimulus, served to promote neural stem cells (NSCs) to differentiate into neurons. This was observed through the systematic investigation of the effect of the persistent photoconductivity (PPC) of GaN on the differentiation of primary NSCs from the embryonic rat cerebral cortex. NSCs were directly cultured on the GaN surface with and without ultraviolet (UV) irradiation, with a control sample consisting of tissue culture polystyrene (TCPS) in the presence of fetal bovine serum (FBS) medium. Through optical microscopy, the morphology showed a greater number of neurons with the branching structures of axons and dendrites on GaN with UV irradiation. The immunocytochemical results demonstrated that GaN with UV irradiation could promote the NSCs to differentiate into neurons. Western blot analysis showed that GaN with UV irradiation significantly upregulated the expression of two neuron-related markers, βIII-tubulin (Tuj-1) and microtubule-associated protein 2 (MAP-2), suggesting that neurite formation and the proliferation of NSCs during differentiation were enhanced by GaN with UV irradiation. Finally, the results of the Kelvin probe force microscope (KPFM) experiments showed that the NSCs cultured on GaN with UV irradiation displayed about 50 mV higher potential than those cultured on GaN without irradiation. The increase in cell membrane potential may have been due to the larger number of photogenerated free charges on the GaN surface with UV irradiation. These results could benefit topical research and the application of GaN as a biomedical material integrated into neural interface systems or other bioelectronic devices.

## 1. Introduction

GaN, as a wide-bandgap semiconductor, has been widely used in power devices, microwave RF devices, and optoelectronic devices because of its high thermal conductivity, high breakdown field strength, high saturated electron drift rate, and high photoelectric conversion efficiency. With the continuous expansion of the application of GaN, its applications in the biomedical field, such as in biosensors [1,2,3,4], nanoscale drug carriers [5], microelectrode arrays [6,7], and functional device interfaces for the detection of cells [8,9,10], have attracted increasing interest owing to its good physical and chemical stability, providing a platform for the study of the effects of semiconductors on cell behavior. In addition, GaN can be easily integrated into existing electronic and photoelectronic semiconductor manufacturing processes. Thus, it is necessary to develop a biological interface with electronic activity and stability that is conducive to cell growth based on GaN substrates. Neural cells, due to their high electrical sensitivity [11], have shown potential in connecting biological systems combined with semiconductors [9,12,13,14,15]. It has been proven that GaN semiconductors can mediate cell reactions in space and promote the attachment, differentiation, and neurite growth of neuron cells. Living rat pheochromocytoma cells (PC12) provide a mature neuron model system in which one can identify exposure to nerve growth factor (NGF) [16]. Jewett, S.A., et al. explored the behavior of PC12 cells on the surface of GaN and found that GaN, with its biocompatibility and non-toxicity, could offer a candidate for the detection of chemical and electrical stimuli related to neural interfaces [17]. Young T H et al. found that neurons cultured on a GaN substrate formed a dense fiber neural network [12,13]. The excellent biocompatibility [17,18,19], chemical stability [20,21,22], and electrical properties in ionic solutions [1,14,23] of GaN indicate that it could be a promising material for interfacing with nerve cells in the regenerative medicine field.

Primary neural stem cells (NSCs) are emerging as a potential regeneration and therapeutic strategy to treat a wide variety of neurological diseases [24,25]. NSCs have the ability to self-renew and differentiate into both neurons and astrocytes to form mature brain tissue. Many efforts have been made to investigate the influence of culture substrates in regulating the directional differentiation of NSCs, such as those focusing on the morphology [26,27,28,29,30] and stiffness [31,32,33] of substrates. Chen, C.R., et al. studied the effect of a GaN substrate on differentiation and provided evidence that GaN could induce NSCs to differentiate into neurons [13,34]. However, the possible reasons for such induced differentiation of GaN were not given. More recently, investigations of NSCs cultured on semiconductors (e.g., SiC and Si [35], graphene [36,37,38,39], Cu_2_S [40], carbon dots [41], and diamond [42]) have been reported, in which the biosafety and biocompatibility of the semiconductors have been studied in detail, and the effects on the differentiation of NSCs, other cells, and proteins have also been mentioned.

**Scheme 1:**
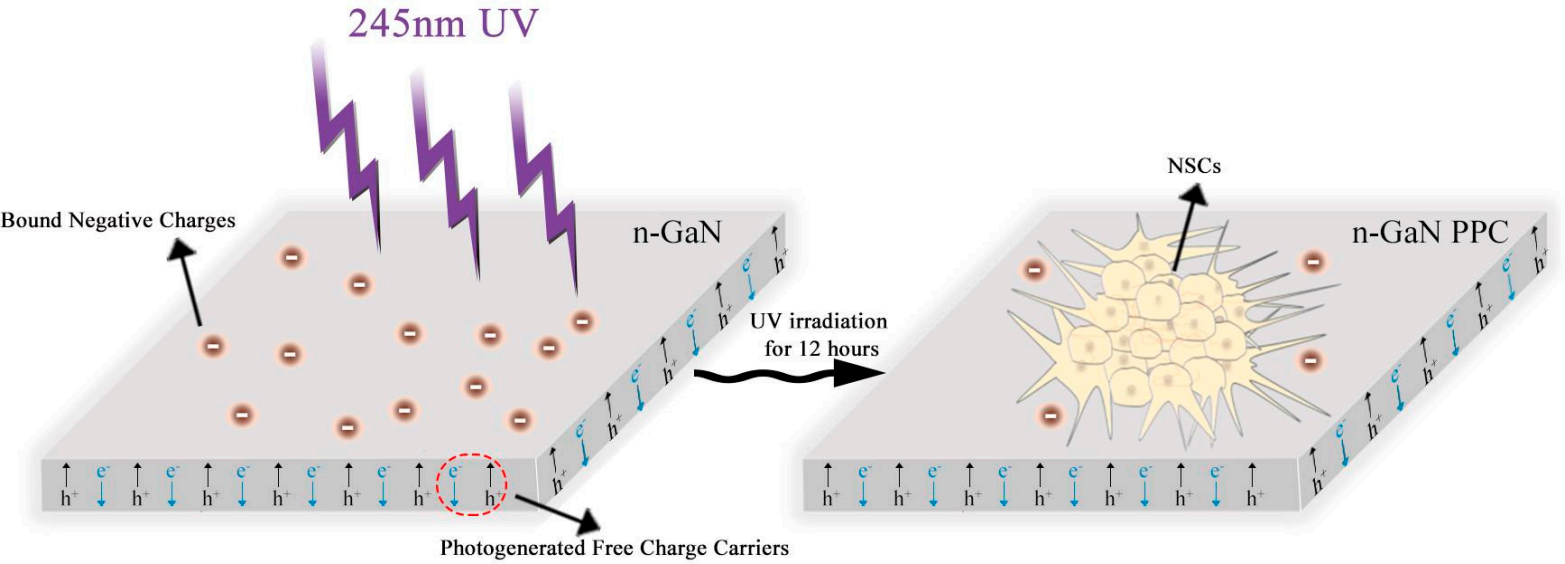
Representation of the PPC approach to generating a photogenerated charge to stimulate NSCs in vitro.

In this work, we found that the photogenerated charge of a GaN substrate, due to its PPC properties (Scheme 1), as an exogenous stimulus, could promote NSCs to differentiate into neurons. The surface of GaN was irradiated with UV light to realize PPC and generate free charge carriers on the GaN surface, and then NSCs were cultured on it. For comparison, GaN without UV irradiation and TCPS were also used to culture NSCs under the same conditions. The morphology, examined through the optical microscope, showed a greater number of neurons with the branching structures of axons and dendrites on GaN with UV irradiation. Immunocytochemical characterization showed that GaN with UV irradiation could promote the NSCs to differentiate into neurons, as compared to GaN without UV irradiation and TCPS. Western blot analysis showed that Tuj-1 and MAP-2, two markers for early and mature neurons, were both expressed at a higher level. KPFM measurements revealed that the surface potential of the NSCs on GaN increased by approximately 50 mV under UV irradiation compared to those on GaN without UV exposure. This indicates an increase in cell membrane potential, likely due to the process of PPC. The reason for which GaN induces NSCs to differentiate into neurons was experimentally proven to be the free charge carriers on the GaN surface, as an exogenous stimulus, using the PPC process. Therefore, GaN could be a novel model system for the regulation of neuronal behavior in semiconductor biointerfaces, in line with the development of applications involving the use of GaN in neurochips, biosensors, and implantable bioelectronic devices.

## 2. Results and Discussion

### 2.1. Surface Potential of GaN after UV Irradiation

**Figure 1:**
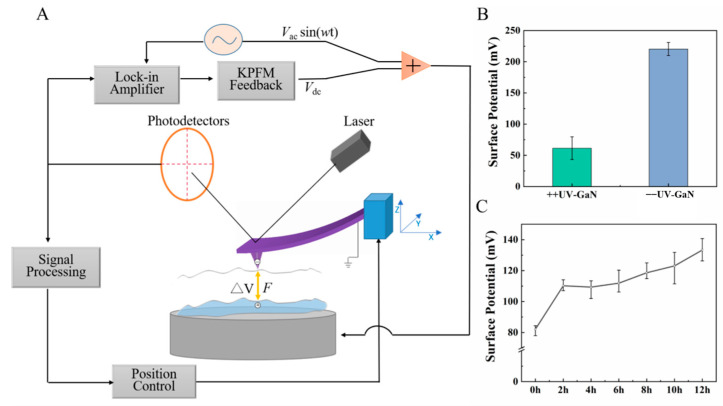
(**A**) Schematic of KPFM measurement with a metal-coated tip, (**B**) GaN surface potential before and after exposure to UV light, and (**C**) changes in surface potential as a function of time after illumination with UV light.

GaN has a wide bandgap of 3.4 eV, and, due to its material properties, it can only be excited by UV irradiation. Moreover, GaN is a semiconductor with PPC and can produce instantaneous photo-induced free carriers under UV illumination, showing an increase in conductivity that can last for hours or days [21,43]. Due to the upward band bending of *n*-GaN, a space charge region is formed on its surface, which is negatively charged on the surface and positively charged inside, forming an electric field pointing to the surface. When UV light irradiates GaN, electron-hole pairs are generated and separated under the electric field, and the electrons drift to the inside of GaN, while the holes migrate to the surface. Therefore, the reduction in upward band bending and the decrease in the surface potential of GaN are caused by the accumulation of photogenerated carriers in the depletion layer [44,45,46]. KPFM is an effective method to obtain the surface potential, which represents the difference between the work function of the sample minus the work function of the AFM probe. Higher surface potential is closely linked with a higher work function. Figure 1A shows a schematic of the KPFM setup employed for our experiments. As is shown in Figure 1B, the surface potential of GaN was reduced from 220.3 ± 10.5 mV to 61.5 ± 18.3 mV before and after exposure to UV light. After removing the UV light, the surface potential underwent a stable period for about 6 h and then gradually and slowly recovered to the initial surface potential of GaN without UV irradiation (Figure 1C).

### 2.2. Effect of GaN on the Differentiation of NSCs 

**Figure 2:**
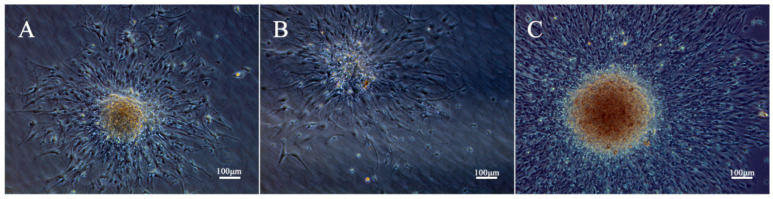
The morphology of NSCs from an optical microscope, cultured on (**A**) GaN with UV irradiation, (**B**) GaN without UV irradiation, and (**C**) TCPS after 3 days of incubation.

**Figure 3:**
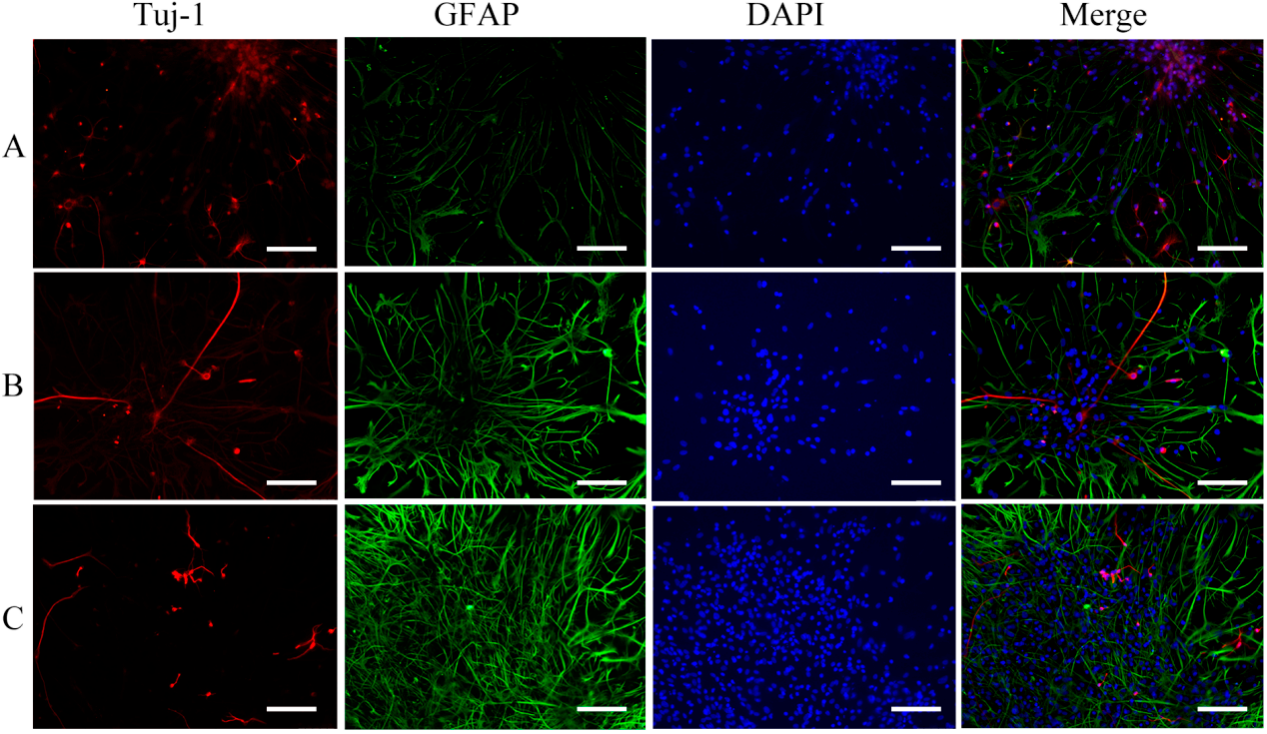
Immunohistochemical analysis of neuronal and astrocyte markers on (**A**) GaN after UV irradiation, (**B**) GaN without UV irradiation, and (**C**) TCPS (scale bar = 100 μm). Neurons were characterized by Tuj-1, astrocytes by GFAP, and nuclei by DAPI as controls.

The properties of the NSCs were confirmed by immunofluorescence and flow cytometry, as shown in Figures S2 and S3, before being cultured on the samples. Figure 2A–C shows the morphology of the NSCs as obtained from an optical microscope, cultured on GaN with UV irradiation, GaN without UV irradiation, and a TCPS substrate, respectively, under the conditions of an induction medium (98% DMEM/F12, 1% FBS, 1% penicillin/streptomycin) after 3 days of incubation. It can be seen that the NSCs were induced to differentiate into a greater number of neurons with the branching structures of axons and dendrites on GaN with UV irradiation, as compared to GaN without UV irradiation. Meanwhile, the NSCs on the TCPS were induced to differentiate into a large number of astrocytes with long processes radiating from the central cell body. However, the effect of the substrate on differentiation cannot be determined only from the morphology, because NSCs often differentiate into a heterogeneous population involving neuronal and astrocytic cells. Thus, an immunocytochemical approach was adopted to evaluate the number of different phenotypes after the NSCs were cultured for 7 days on GaN with UV irradiation, GaN without UV irradiation, and a TCPS substrate, respectively, by using antibodies of Tuj-1 and GFAP (Figure 3). The immunohistochemical analysis showed that more Tuj-1-positive neurons were located on GaN with UV irradiation, compared with the other two. A great number of GFAP-positive astrocytes with extending processes and relatively fewer neurons were observed on the TCPS substrate. The number of neurons on GaN without UV irradiation seemed to be close to that on TCPS. These results reveal that the PPC properties of GaN can promote NSCs to differentiate into more neurons, indicating that there could be a direct relation between the surface electrical properties of GaN and the differentiation direction of NSCs.

**Figure 4:**
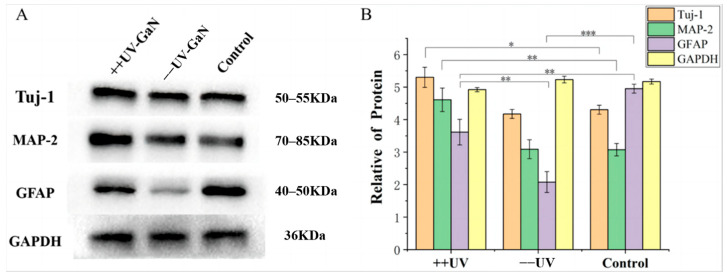
(**A**) Western blots performed with Tuj-1, MAP-2, and GFAP proteins on NSCs cultured for 7 days on GaN with and without UV irradiation and TCPS, (**B**) The relative intensities of Tuj-1, MAP-2, and GFAP levels, determined by band densitometry analysis. Data normalized to the housekeeping gene GAPDH. Data are presented as mean ± SD for triplicate experiments. * *p* < 0.05; ** *p* < 0.01; *** *p* < 0.001.

Tuj-1 and MAP-2 have often been used as early and mature markers of neuronal lineages in vivo. Therefore, a Western blot analysis was carried out to quantitatively evaluate the effects of the substrates on the levels of Tuj-1 and MAP-2 expression after 7-day culture. Figure 4 shows that the expression of the Tuj-1 and MAP-2 proteins related to neurons was obviously upregulated by GaN with UV irradiation, but the induction of the GFAP protein related to astrocytes was not as strong as that on the TCPS substrate. The expression levels of the Tuj-1 and MAP-2 proteins on GaN without UV irradiation were almost the same as those on TCPS, while the expression level of the GFAP protein was the lowest among the three substrates. These results indicate that the differentiation of NSCs on the surface of GaN with UV irradiation can enhance the formation and growth of synapses. 

### 2.3. Characterization of the Surface Potential of NSCs on GaN with KPFM

**Figure 5:**
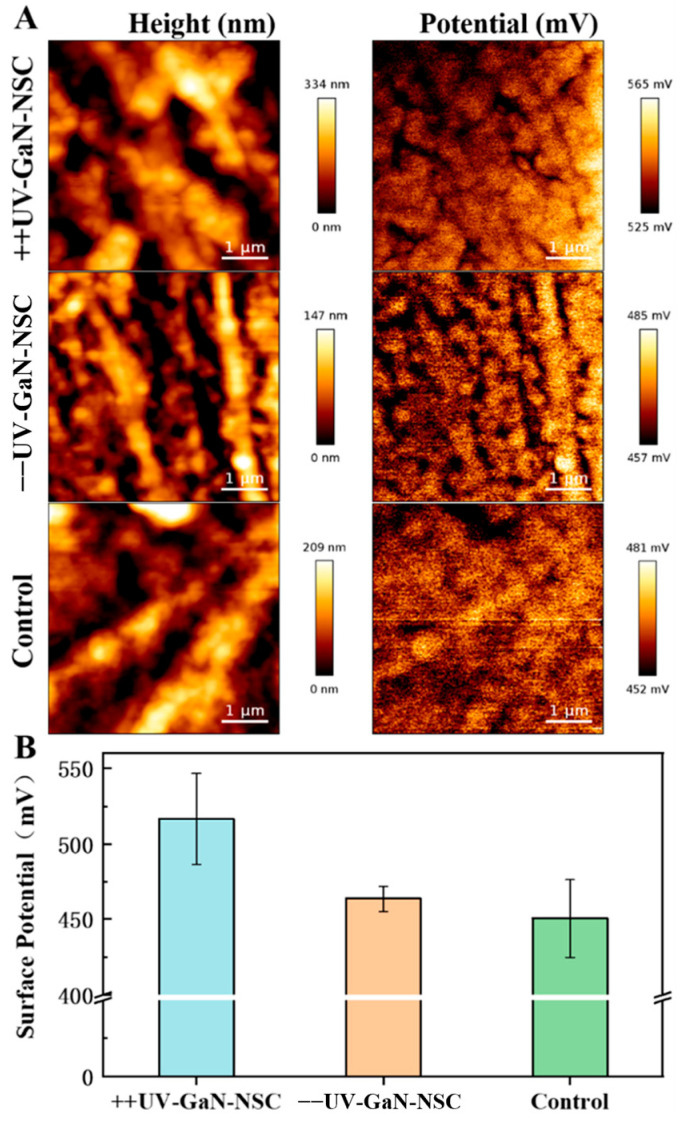
KPFM measurement of the morphology and surface potential distribution of NSCs cultured on GaN with and without UV irradiation. (**A**) Morphology (**left**) and surface potential (**right**). (**B**) Statistical values of surface potential.

**Figure 6:**
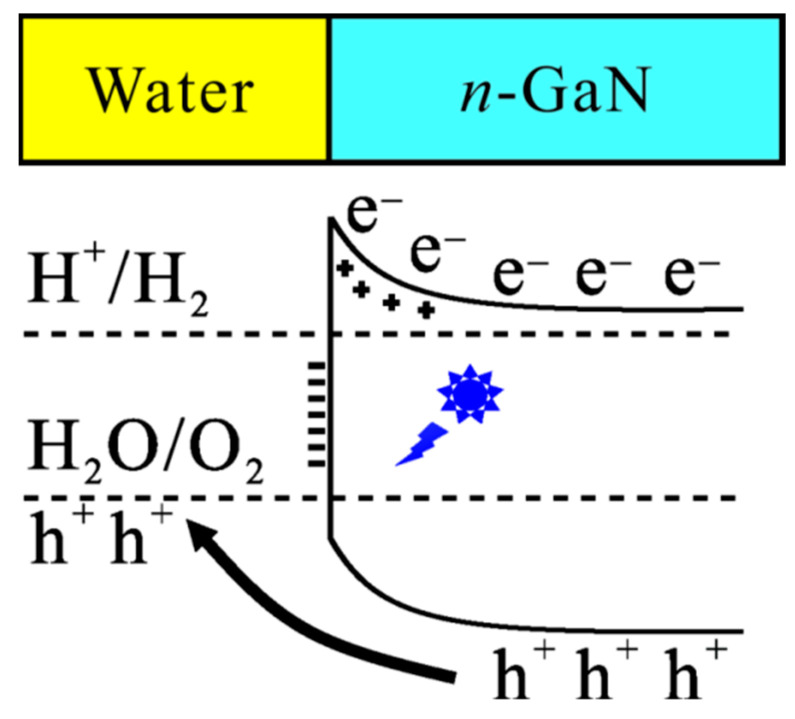
The band structure of water and *n*−GaN connected into one system.

In order to further study the effects of the electrical characteristics of the GaN surface on NSC differentiation, we measured the surface potential distribution of NSCs cultured on GaN with and without UV irradiation and TCPS by KPFM for 7 days. The measured cells were dehydrated and dried, and the measurement was carried out in air at standard ambient temperature (25 °C). Studies have shown that the surface potential of dried biomaterials (such as proteins/DNA) is close to that obtained by other methods in a liquid [47]. Therefore, the results from dehydrated cells can also reflect the electrical characteristics of cells under physiological conditions [48]. We measured the neural network and surface potential on the surfaces of the samples using KPFM. As shown in Figure 5A, it was found that the morphology formed by differentiation was highly consistent with the potential distribution, and the potential distribution was relatively uniform. The statistical analysis of the surface potential was carried out based on ten different regions measured on the samples. As shown in Figure 5B, it was found that the surface potentials of the neural network on GaN with and without UV irradiation and TCPS were 517 ± 30 mV, 465 ± 9 mV, and 456 ± 26 mV, respectively. The PPC process caused by UV illumination will produce electron–hole pairs. For *n*-GaN, the energy band bends upward on the surface, resulting in bound negative charges on the surface, while positive charges will appear in the body, thus forming a space charge region near the surface. Moreover, the electron–hole pairs will be separated under the electric field in the space charge region. Positively charged holes drift to the surface, while negatively charged electrons move to the body. However, unlike the original bound negative charges on the surface, the positive holes migrating to the surface are free carriers. When GaN is in the cell culture medium, according to the energy band relationship between GaN and H_2_O/O_2_ shown in Figure 6, the valence band of GaN is lower than the energy level of H_2_O/O_2_. Therefore, the positive holes on the surface will probably enter H_2_O/O_2_, thus forming an exogenous stimulus for cell differentiation. As is known, the generation and propagation of neural signals are carried out in the form of ions. In addition, it has been reported that electrical stimulation affects the voltage-gated Ca^2+^ channel current, synaptic connection, and excitatory nerve transmission in neural networks. Therefore, the positively charged free holes generated by PPC will provide a microenvironment with exogenous electrical stimulation for cell differentiation, and the free holes attached to the cell membrane surface will raise the work functions of the cell membranes, resulting in an approximately 50 mV increase in the surface potential of the nerve cells on GaN with UV irradiation, compared to that on GaN without UV irradiation. However, the charges on the surface of the GaN without UV irradiation were bound charges, which had little effect on the cell membranes, so the surface potential was almost the same as that for TCPS. In addition, the free holes may even enter the NSCs to directly enhance the spontaneous Ca^2+^ oscillation and postsynaptic current and promote the generation and conduction of nerve signals, thus further promoting the differentiation of NSCs into neuron cells.

## 3. Materials and Methods

### 3.1. Sample Preparation

Here, *n*-type GaN (*n*-GaN) substrates with a size of approximately 10 mm × 10 mm and a 350 μm thickness were grown using hydride vapor phase epitaxy (HVPE) equipment from Suzhou Nanowin Science and Technology Co., Ltd., Suzhou, China (NANOWIN). All culture substrates were cleaned by sonication in deionized water 3 times and for 1 min each and in absolute alcohol (concentration ≥ 99.7%) for 5 min, sterilized in an autoclave, and then rinsed extensively with phosphate buffer solution (PBS). Subsequently, GaN was irradiated with UV light (254 nm, 75 uW/cm^2^) for 12 h on an ultra-clean table and then placed in one well of a 6-well TCPS plate (Corning, New York, NY, USA) for NSC culture. GaN without UV irradiation was also placed into a well of the TCPS plate, and an empty well was used as a control. 

### 3.2. Isolation and Culture of NSCs

NSCs were extracted from SD rat embryos at around 13–15 days of pregnancy. In D-Hanks solution, tissue from both cerebral hemispheres of the fetal rats was removed on ice, and the leptomeninges and blood vessels were removed. Then, the tissue was transferred to a serum-free medium (95% DMEM/F12, 2% 1XB-27, 1% (20 μg/mL) bFGF, 1% (20 μg/mL) EGF growth factor, 1% penicillin/streptomycin) and cut into approximately 1 mm^3^ fragments. After the tissue was collected by centrifugation (1000 r/min, 2 min), it was digested using 1 mL of Accutase™ (Gibaco, Waltham, MA, USA) enzyme solution at 37 °C for 10 min, with gentle shaking every 2 min to ensure uniform digestion, and the cells were collected by filtration. The cells were counted at a density of 1 × 10^6^ cells/mL on a blood cell count board and re-suspended in the serum-free medium. The extracted cells were cultured in T25 culture flasks (Corning, USA) at a temperature of 37 °C, humidity of 95%, and air of 5% CO_2_. After 1 day of culture, the suspension cells were subjected to cell proliferation division and then single cells gradually aggregated. Cell division continued for an additional 3 days, and then the proliferating cells formed neurospheres. Subsequently, the neurospheres were inoculated onto GaN with and without UV irradiation and TCPS. The effect of each substrate on the NSCs was observed in the conditions of the induction medium (98% DMEM/F12, 1% FBS, 1% penicillin/streptomycin). As shown in Figure S1, the morphologies of the NSCs in different periods were observed using an inverted microscope (Leica DMi8, Wetzlar, Germany).

### 3.3. Immunocytochemistry

Well-grown NSC neurospheres were inoculated at a density of 1 × 10^4^ cells/mL in a Petri dish coated with 0.1 g/L poly-lysine. The cells were cultured for 12 h to ensure that the NSCs adhered to the wall. The expression of neuronal marker protein Tuj-1 and astrocyte marker protein GFAP after NSC differentiation was identified. The cultured cells were fixed in 40 g/L paraformaldehyde solution for 15 min. The cells were then treated with 0.5% TritonX-100 for 10 min and blocked with 10% goat serum by volume fraction for 15 min for immunocytochemical identification. Cells were incubated overnight (12 h) at 4 °C in primary antibodies diluted in 200 μL PBS and washed three times with PBS, with each wash lasting 5 min. The primary antibodies and dilutions used in this study were mouse monoclonal anti-Nestin, mouse monoclonal anti-β-tubulin III (anti-Tuj1), and rabbit polyclonal anti-GFAP (1:200, Proteintech Group, Chicago, IL, USA). Next, 200 μL secondary antibody, CoraLite594-conjugated goat anti-mouse IgG (H + L), and CoraLite488-conjugated goat anti-rabbit IgG (H + L) (1:500, Proteintech Group, USA) were incubated separately. The cells of the genus and species of the second antibody and its corresponding antibody were incubated for 2 h at room temperature. Finally, 4′, 6-diamidino-2-phenylindole (DAPI) was used as a nuclear counterstain. Immunohistochemically stained cells were observed with an inverted fluorescence microscope (Leica DMi8, Wetzlar, Germany).

### 3.4. Flow Cytometry Detection

In the context of NSCs, CD133 is expressed, while other markers, such as CD34 and CD45, are not. This distinct expression profile highlights the importance of CD133 in identifying and characterizing NSCs. The neurospheres of NSCs with good growth conditions were collected by centrifugation. The NSCs were digested into a single-cell suspension with StemPro Accutase solution and then collected by centrifugation. The count was re-suspended on the EP tube, and the cell density was adjusted to 1 × 10^6^ cells/mL. FITC anti-mouse CD45 and FITC anti-mouse CD34 (both Proteintech Group, Chicago, IL, USA) were added and the specimens were incubated at room temperature under dark conditions for 30 min. The cells in the other EP tube were fixed with 4% paraformaldehyde for 10 min. Then, the cells were stained with CD133 monoclonal antibody and CoraLite 488-conjugated AffiniPure goat anti-mouse IgG (H + L) at a dilution ratio of 1:1000. The EP tube cells were centrifuged and resuspended in PBS and transferred to the flow tube. The samples were examined via flow cytometry (BD FACSCanto II) and the data were analyzed with the FlowJo software (version 18.0.0.0). The negative control featured a single-cell suspension that was devoid of antibodies.

### 3.5. Western Blot Analysis

After 7 days of induced differentiation, the cells were washed with PBS twice. The cells were lysed in ice-cold RIPA cleavage buffer (50:1 lysate–PMSF inhibitor, BOSTER, Wuhan, China) for about 30 min and ultrasonically processed at 4 °C for 15 s. The supernatants were preserved for protein analysis and Western blotting analysis. The protein concentration was determined with a BCA Protein Assay Kit (Solarbio, Beijing, China). The supernatants were added to a 1/5 volume loading buffer (BOSTER, Wuhan, China) and heated to 95 °C for 10 min. Each proteome sample (50 µg of total protein per lane) was separated by SDS-PAGE on a 10% polyacrylamide gel. Initially, electrophoresis was conducted at a constant voltage of 80 V until the samples in the stacking gel were compressed into a narrow band and the rainbow marker showed slight separation. The voltage was then increased to 120 V to continue the electrophoresis until the bromophenol blue indicator line approached the bottom of the glass plate. At this point, electrophoresis was stopped, and the protein gels were transferred to a PVDF membrane at a current of 250 mA for 2 h. The membranes were blocked with 5% non-fat milk in TBST buffer for 2 h (Solarbio, Beijing, China). One mouse monoclonal anti-Tuj1 antibody, rabbit monoclonal anti-MAP2 antibody, and rabbit polyclonal anti-GFAP (1:1000, Proteintech Group, USA) were added and the specimens were incubated for 12 h at 4 °C. 

### 3.6. KPFM Characterization

KPFM measurement was performed with atomic force microscopy (AFM, JPK NanoWizard 4 BioScience, Bruker, Billerica, MA, USA). The probe tip throughout all measurements was the SCM-PIT-V2 with a spring constant *k* of 3.0 N/m and resonant frequency *f*_0_ of 75 kHz. The surface potential difference was characterized using a scan rate of 0.3 Hz with a resolution of 512 × 512 pixels.

### 3.7. Experimental Animals

All animal experimental procedures were conducted in accordance with the guidelines for the care and use of laboratory animals from the National Institutes of Health and were approved by the Jiangxi Experimental Animal Management Committee (SYXK (Gan) 2018-0004).

## 4. Conclusions

We conclude that the PPC of GaN produced photogenerated free charge carriers as an exogenous stimulus to promote NSCs to differentiate into more neurons, as compared with the experimental results obtained for GaN without UV irradiation and TCPS. The reported results are of significance and indicate that, as long as a material can excite free charge carriers under light, it can likely promote the differentiation of NSCs into neurons. This is not limited to GaN, and it will open up new opportunities for the future application of electrical stimulation in the directional differentiation of stem cells or the regulation of cell adhesion, elastic moduli, and signal transduction. At the same time, our work will also help to promote the application of bioelectronic devices based on GaN biointerfaces.

## Data Availability

Data are included in the article and Supporting Information.

## Supplementary Materials

The following supporting information can be downloaded at: https://www.mdpi.com/article/10.3390/molecules29184439/s1, Figure S1: The morphological changes of NSCs in different periods were observed under an inverted microscope. A: 1d; B: 3d; C: 5d; D: 7d; Figure S2: Nestin identification of NSCs marker under an immunofluorescence microscope. A: positive expression of Nestin; B: The expression of DAPI was positive; C: overlap of A and B; Figure S3: The Expression of CD markers on NSCs surface measured by flow cytometry.
