# Supplementary material for: Investigation of Persistent Photoconductivity of Gallium Nitride Semiconductor and Differentiation of Primary Neural Stem Cells
Source: Molecules. 2024 Sep 19;29(18):4439. doi: 10.3390/molecules29184439 (PMC11434078; doi:10.3390/molecules29184439)
Supplement: Supplementary file 1 [file molecules-29-04439-s001.zip › molecules-3141485-supplementary.pdf]

SUPPORTING INFORMATION

Investigation of persistent photoconductivity of gallium nitride semiconductor on the differentiation of primary neural stem cells

Yu Meng [1, 3, 4, †], Xiaowei Du [5 †], Shang Zhou [1, 3, 4], Jiangting Li [1, 3, 4], Rongrong Feng [1, 3, 4], Huaiwei Zhang [1, 3, 4], Qianhui Xu [1, 3, 4],Weidong Zhao [1, 3, 4, *], Zheng Liu [2, *], Haijian Zhong [1, 3, 4, *]

[1] Key Laboratory of Biomaterials and Biofabrication in Tissue Engineering of Jiangxi Province, Gannan Medical University, Ganzhou 341000, P. R. China

[2] School of Materials Science & Engineering, Nanyang Technological University, Singapore 639798, Singapore

[3] School of Medical Information Engineering, Gannan Medical University, Ganzhou 341000, P. R. China

[4] Key Laboratory of Prevention and Treatment of Cardiovascular and Cerebrovascular Diseases, Ministry of Education, Gannan Medical University, Ganzhou 341000, P. R. China

[5] Department of Diagnostic Radiology, First Medical Centre, PLA General Hospital, Beijing 100000, P. R. China

1. Extraction and culture of primary NSCs:

NSCs were extracted from SD rat embryos around 13-15 days pregnant. In the D-Hnanks solution, the tissues of both cerebral hemispheres of fetal rats were removed on ice and the leptomeninges and blood vessels were removed. Then tissues were

[*] Corresponding authors.
Email addresses: zhaowd@gmu.edu.cn (W. Z.); Z.Liu@ntu.edu.sg (Z. L.); hjzhong2007@gmu.edu.cn (H. Z.)
[†] These two authors equally contributed to the work.

transferred to a serum-free medium (95% DMEM/F12, 2% 1xB-27™, 1% (20 μg/mL) bFGF, 1% (20 μg/mL) EGF growth factor, 1% Penicillin/Streptomycin) and cut into 1 mm^3 fragments. After the tissue was collected by centrifugation and digested with enzymes, the cells were collected by filtration. The cells were counted as density $1x10^6$ cells/mL on the blood cell count board and re-suspended in the serum-free medium. The extracted cells were cultured in T25 culture flasks (Corning, USA) at a temperature of 37 °C, humidity of 95%, and air of 5%CO2. After 1 day of culture, the suspension cells were subjected to cell proliferation division. Single cells then aggregate. Cell division continued for an additional 3 days, after which proliferating cells formed neurospheres. The extracted cells were obtained from the brain tissue of about 14-day-old fetal rats. The suspension growth of the cells could be seen on the first day, with different shapes and small volumes. There are a small number of sheets and impurities of different sizes in the culture medium (Figure S1. A). When the culture medium was changed on the third day, the flakes decreased and the impurities became less and less. The suspension cells gather slowly, and a single cell appears and grows in suspension. The cells are spherical or oval (Figure S1. B). And then the cells get bigger. Several nerve balls gather to form a cell mass similar to mulberry, which is more uniform in size and more regular in shape. The cells had no obvious protuberance and good refraction. (Figure S1. C). On the 7th day of culture, the diameter of NSCs was more than 100 μm. The central part of the neuro ball had become opaque, obvious protuberances could be seen around it. And the central light transmittance was very poor (Figure S1. D). At this time, the cells need to be passaged, otherwise, it will prevent the diffusion of cellular nutrients and cell

growth and metabolism in the NSCs neuro bulb.

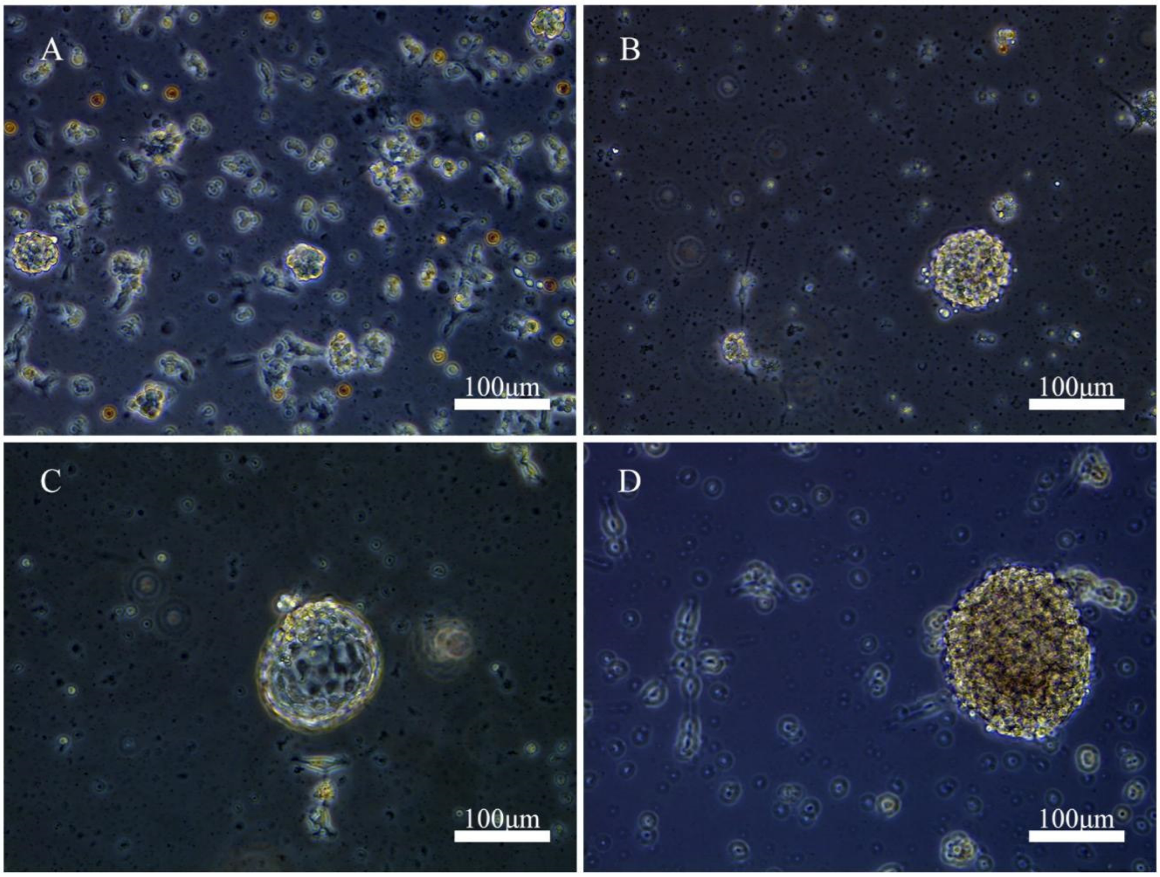

Figure S1. The morphological changes of NSCs in different periods were observed under an inverted microscope. A: 1d; B: 3d; C: 5d; D: 7d.

2. Identification of NSCs:

Nestin was used as the neural stem cell marker protein. Immunofluorescence results showed that after adherent treatment, single cells were gathered, and the nucleus was small and stained blue by DAPI (Figure S2. B). The cytoplasm was green, indicating that Nestin was positive (Figure S2. A). For image acquisition in S2, a total magnification of 80× was used, achieved with a 20× objective lens and a 4× ocular lens. Fluorescence

imaging was performed using specific filter sets: DAPI (EX: 325-375 nm, DC: 400 nm, EM: 435-485 nm), FITC (EX: 460-500 nm, DC: 505 nm, EM: 512-542 nm), and RHOD_LP (EX: 517-563 nm, DC: 580 nm, EM: 590 nm). The excitation sources included DAPI (EX: 325-375 nm), FITC (EX: 460-500 nm), and RHOD_LP (EX: 517-563 nm). Post-acquisition image processing was performed using Leica LAS X software.

CD133, a surface marker of neural stem cells, was detected by flow cytometry. CD133, also known as Prominin-1, is a member of the five-time transmembrane glycoprotein Prominin family. It was initially used as a specific marker to screen stem cells and progenitor cells. NSCs express CD133 but do not express CD34 and CD45. For identifying NSCs, the gating strategy was based on cell surface marker CD133, with the unlabelled single-cell suspension of NSCs used as a negative control for setting the gate position. Bisector gating on the histogram was used to discriminate between positive and negative populations. As shown in the Figure S3, the expression rates of CD133, CD34, and CD45 are 86.8%, 5.09%, and 7.53%, respectively, the control group showed a rate of 0.18%. Therefore, it can be preliminarily concluded that the isolated and cultured NSCs expressed its specific marker (Nestin+, CD133+, CD34-,CD45-), which had the characteristics of the NSCs. The NSCs ware completely suitable for the differentiation of NSCs cells induced by GaN.

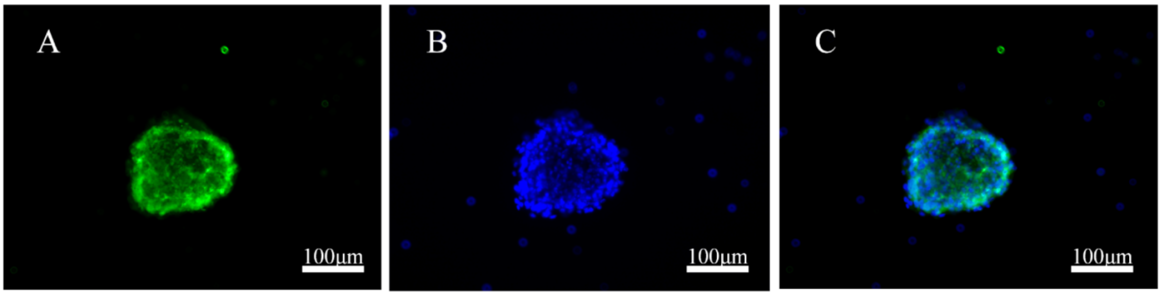

Figure S2. Nestin identification of NSCs marker under an immunofluorescence microscope. A: positive expression of Nestin; B: The expression of DAPI was positive; C: overlap of A and B.

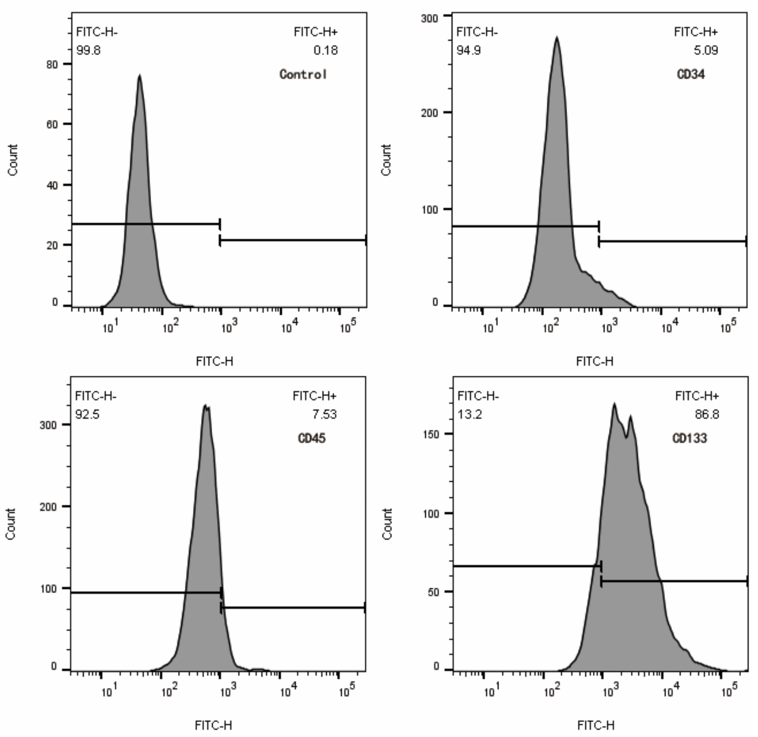

Figure S3. The Expression of CD markers on NSCs surface measured by flow cytometry.
